# Studies on Some New Ru(III) Complexes Using aryl-azo Pentane- 2,4-dione and 2,6-bis (2'-Benzimidazolyl) Pyridine as Ligands: Synthesis, Spectroscopic, Luminescent, Electrochemical and Biological Activities

**DOI:** 10.1155/MBD.2001.65

**Published:** 2001

**Authors:** Lallan Mishra, Ajay K. Yadaw, Ratna S. Phadke, Chang S. Choi, Koji Araki

**Affiliations:** 1 Department of Chemistry Banaras Hindu University Varanasi 221 005 India; 2 Chemical Physics Division TIFR Mumbai India; 3 Institute of Industrial Science University of Tokyo Japan; 4 KAIST Korea

## Abstract

Some ruthenium(III) complexes with aryl-azo 2,4-pentanedione as co-ligands (L^1^H - L^3^H_2_) have been synthesized and characterized spectroscopically IR, ^1^H NMR, UV/Vis, ESR, conductimetric) along with elemental analysis and FAB-mass data. Their luminescent and redox properties have been studied. The antibacterial, anti-HIV and antitmnour activities have also been reported.


### STUDIES ON SOME NEW RU(III) COMPLEXES USING ARYL-AZO PENTANE-2,4-DIONE AND 2,6-BIS (2'-BENZIMIDAZOLYL) PYRIDINE AS LIGANDS: SYNTHESIS, SPECTROSCOPIC, LUMINESCENT, ELECTROCHEMICAL AND BIOLOGICAL ACTIVITIES

Lallan Mishra*[1], Ajay K. Yadaw[1], Ratna S. Phadke[2], Chang S. Choi[#3] and Koji Araki[3]

[1] Department of Chemistry, Banaras Hindu University, Varanasi - 221 005, India
[2] Chemical Physics Division TIFR, Mumbai, India
[3] Institute of Industrial Science, University of Tokyo, Japan

**Abstract**
Some ruthenium(III) complexes with aryl-azo 2,4-pentanedione as co-ligands ($L^1H$ - $L^3H_2$) have been synthesized and characterized spectroscopically (IR, $^1H$ NMR, UV/Vis, ESR, conductimetric) along with elemental analysis and FAB-mass data. Their luminescent and redox properties have been studied. The antibacterial, anti-HIV and antitumour activities have also been reported.

### Introduction

Study of octahedral ruthenium complexes using spectroscopic techniques is of current interest [1-4]. Lahiri *et al.* [5] synthesized several ruthenium(III) complexes of N, O donors and studied their electronic and electrochemical properties. The complexes of $Ru(N\text{-}N)_2Cl_2$ family (where N-N = 2,2'-bipyridine and 1, 10-phenanthroline) have been found [6] unsuitable in view of isomeric problems. Therefore, several research groups have focussed their attention on the complexes of the type $[Ru(tpy)_2]^{2+}$ family (tpy = 2, 2':6', 2"-terpyridine) in spite of the fact that they exhibited less favourable photophysical properties particularly showed weak luminescence at room-temperature with shorter excited-state lifetime. However, suitably substituted terpyridyl complexes especially 4'-substituted terpyridyl Ru(III) complexes were found to display room-temperature luminescence. Thus, in order to avoid isomeric complexities, we selected a terpyridyl type tridentate ligand *viz.* 2,6-bis(2'-benzimidazolyl)pyridine. Thes selectivity of the benzimidazolyl terpyridyl ligand was also based on its involvement in the strong intramolecular stacking interactions between the DNA strands hence in bioactivities [7] of the resulting complexes.

In this context it is also of worth to mention that azo/hydrazo compounds have also been subjected [8] to many biological reactions such as in protein synthesis inhibition, nitrogen fixation and antitumour properties which have been understood in their action as DNA crosslinking agents. Additionally, ruthenium(III) chloro complexes have been reported [9] to bind covalently to calf-thymus DNA. In this context reports [10-11] by Keppler and Sava *et al.* on the tumour inhibiting properties of ruthenium(III) chloro complexes have been found very encouraging.

Thus in view of above mentioned properties and in continuation to our earlier studies [12-14], we found it worthwhile to synthesize, new mono and dinuclear Ru(III) chloro complexes containing aryl diazo-pentane 2,4-dione and 2,6-bis-(2'-benzimidazolyl) pyridine as ligands and to study their spectroscopic, electrochemical, luminescent and antibacterial/antitumour/anti-HIV properties.

### Materials and Methods

All the solvents purchased from E. Merck were distilled using standard procedure prior to use. Pentane2,4-dione, aniline, p-phenylenediamine, benzidine, $RuCl_3.3H_2O$, 2,6-pyridine dicarboxylic acid purchased from Sigma-Aldrich were used as supplied whereas 2,6-bis(2'-benzimidazolyl) pyridine was prepared by a reported [15] procedure. Tetrabutyl ammonium bromide taken from Merck was converted into tetrabutyl ammonium perchlorate (TBAP) by an available procedure [16]. Caution! TBAP could be explosive so the use of small amounts of it are recommended. Neutral alumina for column chromatography was supplied by E. Merck and used as such. All the reactions were carried out under $N_2$ atmosphere.

### Present address. KAIST, South Korea

Microanalysis (C, H and N) performed on a Carlo Erba Elemental Analyzer 1108 and FAB-mass data using a JEOL SX-102 mass spectrometer were carried out at the Central Drug Research Institute Lucknow, India. IR (KBr pellets) and UV/Vis data were obtained using a JASCO FT IR 5300 spectrometer and a Shimadzu UV-1601 spectrophotometer whereas ESR spectra in the solid state as well as in solution (DMSO) at room temperature and liquid $N_2$ temperature were recorded at the Indian Institute of Technology, Mumbai, India. Electrochemical measurements were made using an electrochemical interface SI1287 potentiostate, using a graphite disc as working electrode, platinum wire as auxiliary electrode and $Ag/Ag^+$ as reference electrode in a three electrode configuration. Luminescent spectra were recorded at the University of Tokyo, Japan using a Shimadzu RF 5300 spectrophotometer at 25°C. The antibacterial study was carried out at the School of Biotechnology, B.H.U., Varanasi, India whereas antitumour and anti-HIV activities were evaluated at the School of Pharmacy, University of North Carolina, USA.

**Synthesis of ligands**

The ligands ($L^1H$ - $L^3H_2$) (scheme 1) were synthesized and characterized using IR, $^1H/^{13}C$ NMR, $^1H$-$^{13}C$ HMBC (heteronuclear multiple bond correlation spectroscopy) and FAB-mass data as reported [12] by us whereas 2,6-bis(2'-benzyimidazolyl) pyridine was prepared from 2,6-pyridine dicarboxylic acid and o-phenylenediamine in acidic media following the reported procedure [15].

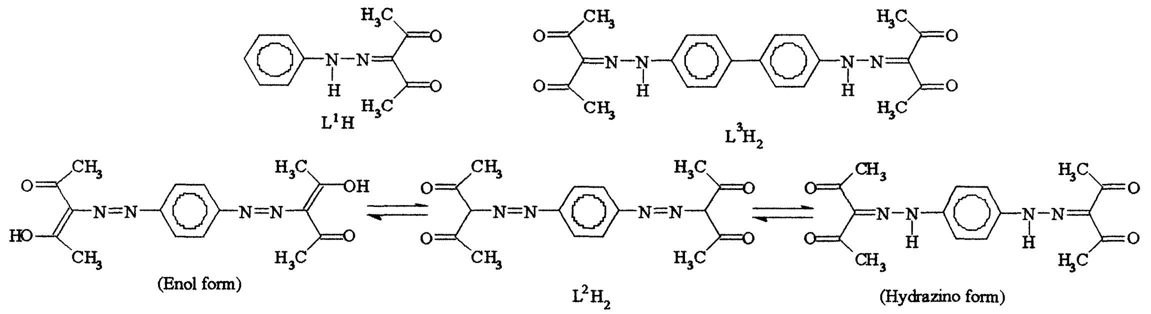

**Scheme 1 : Structure of ligands**

Table I: Physical and analytical data of Ru(III) complexes

| Complex FAB-mass data Calc. (Found) | Yield, % (colour) | Analysis calc. (Found) %C | %H | %N | % $Cl^-$ Calc. (Found) | Λ ($\Omega^{-1}$ $cm^2$ $mol^{-1}$) |
|---|---|---|---|---|---|---|
| $[Ru(L^1H)_3]3Cl^-$ 784.5 (784) $[M-Cl^-]^+$ | 50 (Dark brown) | 46.26 (46.08) | 4.67 (4.54) | 9.81 (9.69) | 14.82 (14.65) | 280 |
| $[Ru_2(L^2H_2)Cl_6(H_2O)_2]$ 781 (781) $[M]^+$ | 55 (Light brown) | 24.58 (24.82) | 2.82 (2.95) | 7.17 (7.08) | 27.27 (26.96) | 28 |
| $[Ru_2(L^3H_2)Cl_6(DMSO)_2]$ 977 (977) [M] | 62 (Redish brown) | 31.93 (31.65) | 3.48 (3.40) | 5.73 (5.62) | 21.81 (21.50) | 36 |
| $[Ru_2(L^1H)(Hbbip)DMSO](PF_6)_2$ 838 (838) $[M-PF_6]^+$ | 58 (Light brown) | 39.06 (39.46) | 3.05 (3.18) | 9.96 (9.85) | - | 224 - |
| $[Ru_2L^2H_2(Hbbip)_2Cl_2](PF_6^-)_2$ 1223 (1222) $[M-2PF_6^-]^{2+}$ | 60 (Dark brown) | 42.82 (42.95) | 2.77 (2.84) | 12.95 (12.78) | 4.69 (4.86) | 236 |

**Synthesis of complexes**

$[Ru(L^1H)_3]$ $3Cl^-$ 1: An aqueous ethanolic solution (10 mL, 1:1 v/v) of $RuCl_3.3H_2O$ (3mM, 0.784g) was mixed to an ethanolic solution (10 mL) of the free ligand $L^1H$ (3 mM, 0.612g) in 1:1 molar ratio while stirring and the resulting mixture was refluxed for 28 hours. The progress of the reaction was monitored using TLC. The volume of the solvent was reduced to half which was then kept in refrigerator for overnight. The brown precipitate thus obtained was filtered and washed with $H_2O$, EtOH followed by $Et_2O$ and then dried *in vacuo*.

[$Ru_2(L^2H_2)$ $Cl_6.2H_2O$] **2** and [ $Ru(L^3H_2)$ $Cl_6$ $(DMSO)_2$] **3** were also prepared as reported above by refluxing separately an aqueous ethanolic solution (10 mL, 1:1 v/v) of $RuCl_3.3H_2O$ (6 mM, 1.575g) with an ethanolic solution (10 mL) of the corresponding free ligands $L^2H_2$ [(3mM, 0.99g) in aqueous ethanol (10 mL, 1:1 v/v)] and ($L^3H_2$) [(3 mM, 1.218g) in DMSO (15 mL)], respectively for 24 hours.

[$Ru(L^1H)$ ($H_2$bbip) DMSO] $(PF_6)_2$ **4**: To the solution of **1** (0.1 mM, 0.820g) in DMSO (10 mL) a hot ethanolic solution (10 mL) of free ligand ($H_2$bbip) (0.1 mM, 0.311g) was added while stirring and the resulting solution was refluxed for 25 hours then cooled at room temperature and filtered. The clear filterate thus obtained was precipitated by the addition of saturated aqueous solution of $NH_4PF_6$. The reddish-brown precipitate thus obtained was filtered and washed with $H_2O$, EtOH and $Et_2O$ successively and finally dried *in vacuo*.

Similarly [$Ru_2(L^2H_2)$ $(H_2bbip)_2$ $Cl_2$] $(PF_6)_2$ **5** was also prepared by the addition of a solution of **2** (0.1 mM, 0.781g) in DMSO (10 mL) to a hot ethanolic solution (10 mL) of the ligand ($H_2$bbip) (0.2 mM, 0.622g) while stirring. After refluxing for 30 hours the solution was cooled at room temperature and then filtered and the solid complex as $PF_6^-$ salt was obtained by the addition of saturated aqueous solution of $NH_4PF_6$ which was isolated and purified as described for **4**.

The elemental analysis and FAB-mass data alongwith other properties of the complexes are shown in Table I.

**Results and Discussion**

The composition of the complexes assigned on the bases of their elemental (C, H and N) analysis and FAB-mass data is shown in Table I. The complexes were found thermally stable at room temperature. Complexes **1**, **4** and **5** were soluble in acetone, acetonitrile, DMF and DMSO whereas **2** and **3** were soluble only in DMF and DMSO. The molar conductance of the complexes shown in Table I is in consistence with the number of counteranions present in the complexes [12,17].

**IR Spectra**: In the IR spectra (KBr) of the complexes, peaks observed at 1620 - 1640 $cm^{-1}$ due to νC=O were found to be lower as compared to free ligands values observed at 1674-1687 $cm^{-1}$. This indicated the participation of both the >C=O groups in the bonding with ruthenium. However in complexes **3**, **4** strong peak observed at 1102-1108 $cm^{-1}$ was assigned to S-coordinated DMSO in view of an earlier report [18]. A strong and sharp peak observed at 839-840 $cm^{-1}$ in the spectra of the complexes **4** and **5** was assigned to $\nu(PF_6^-)$.

$^1H$ NMR spectra: To get further structural support from $^1H$ NMR spectra, one representative complex [$Ru(L^1H)_3$] $3Cl^-$ was reduced into the Ru(II) form in the presence of N-ethyl morpholine using a reported procedure [19]. The $^1H$ NMR spectrum recorded in DMSO-$d_6$ showed two peaks at δ 2.6 and 2.9 ppm due to methyl protons, a complex pattern at δ 7-8 ppm due to phenyl protons and a singlet at δ 14.20 ppm due to the NH proton.

Table II: UV/Vis and Luminescent data of Ru(III) complexes in DMF ($10^{-5}$M) solution

| Complex | $\lambda_{max}$, nm | ($10^{-3}\varepsilon$, $M^{-1}cm^{-1}$) | $\lambda_{em}$, nm |
|---|---|---|---|
| | 269 | (21.3) | 460.00 |
| **1** [$Ru(L^1H)_3$]$3Cl^-$ | 357 | (22.9) | |
| | 593 | (4.72) | |
| | 265 | (21.50) | 436.00 |
| **2** [$Ru_2(L^2H_2)Cl_6(H_2O)_2$] | 395 | (15.16) | |
| | 550 | (8.42) | |
| | 267 | (33.40) | 555.00* |
| **3** [$Ru_2(L^3H_2)Cl_6(DMSO)_2$] | 423 | (67.70) | |
| | 604 | (2.00) | |
| | 272.50 | (17.40) | 370, 719 |
| **4** [$Ru_2(L^1H)$(Hbbip)(DMSO)] $(PF_6)_2$ | 342.50 | (32.70) | |
| | 590.00 | (2.00) | |
| | 269.50 | (50.20) | 375, 725 |
| **5** [$Ru_2(L^2H_2)(Hbbip)_2Cl_2$] $(PF_6)_2$ | 334.50 | (40.30) | |
| | 540.00 | (4.00) | |

$\lambda_{ex}$ was 330 nm except in case of **3** where excitation wavelength was 430 nm.

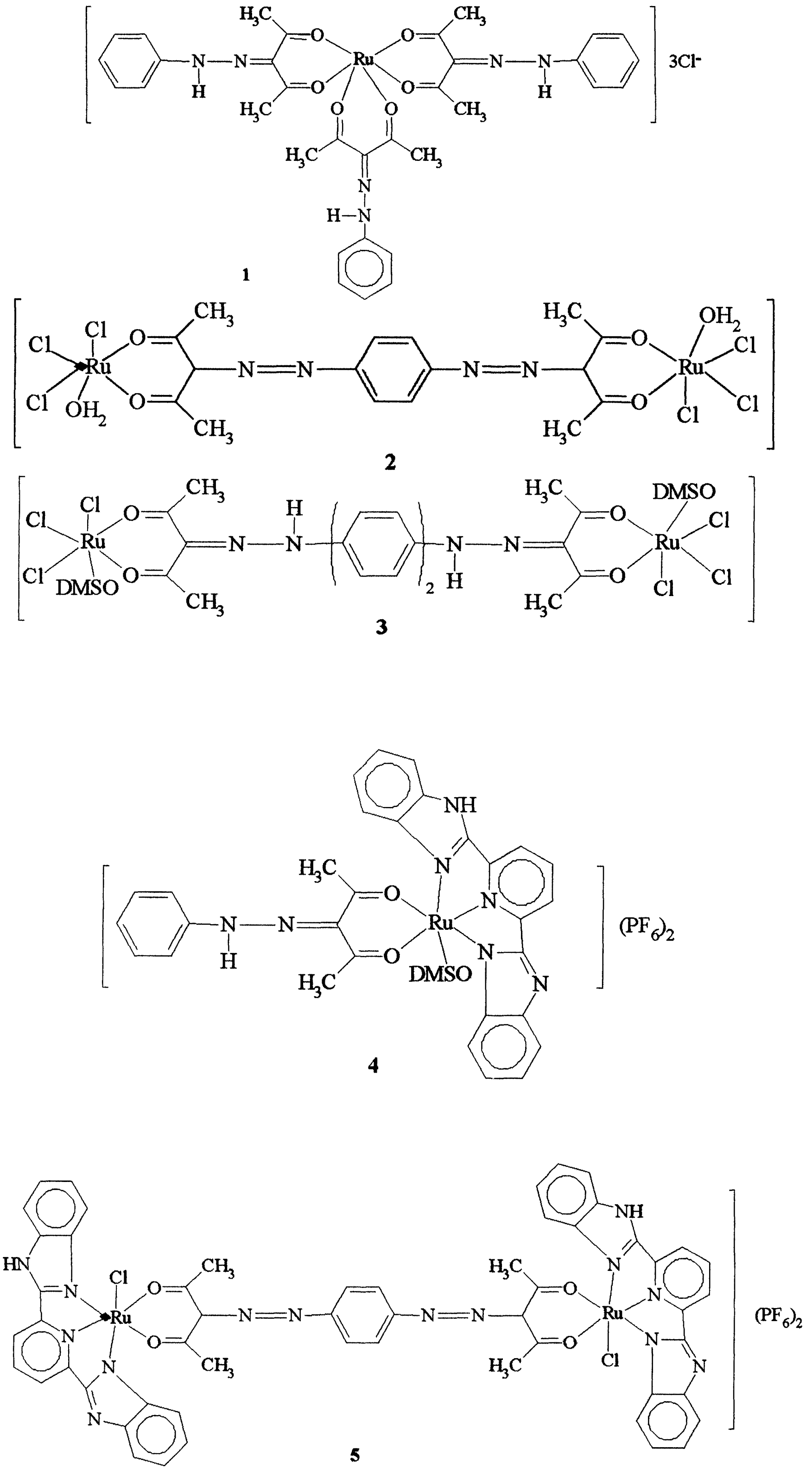

**Figure 1**: Proposed structure of Ru(III) complexes.

**UV/Visible spectra**: UV/Vis spectra of the ruthenium(III) complexes in DMF solution ($10^{-5}$M) were recorded in the region 200-800 nm and the spectral data are listed in Table II.

The data reported in Table II show mainly three transitions in the range 265-272; 334-423 and 540-604 nm. The former two transitions in the higher energy regions were considered to be ligand centered (LC) and latter transition in the range 540-604 nm was assigned to ligand ($\pi^*$) to metal ($t_2^5g$) transition in view of earlier reports [4, 5] on ruthenium(III) systems. Substitution of chloro groups in the complex **2** by 2,6-bis(2'-benzimidazolyl) pyridine lowered the LMCT transition wavelength by 10 nm which is also consistent with the earlier report [20]. Almost double intensity of the peak observed for complex **5** as compared to complex **4** could be considered due to the dinuclear nature [21] of the complex **5**.

**ESR Spectra**: ESR spectra of the complexes were recorded in solid and solution (DMSO) states at room temperature and liquid $N_2$ temperature. The ESR spectrum of the dinuclear complex $[Ru_2(L^2H_2)\,Cl_6(H_2O)_2]$ in the solid state shows six well resolved signals. The g values calculated as 1.80-2.50 were found to lie in the range as reported earlier [22,23]. All the peaks were observed with equal spacing and equal intensity. However for the complexes **1** and **3** a broad spectrum was observed in the solid state at 298 K and 77 K temperatures but in solution (DMSO) they showed three signals and g values, again calculated to be in the range 1.9-2.5.

Thus on the basis of spectroscopic data (IR, $^1$H NMR, UV/Vis, ESR, conductimetric) alongwith elemental analysis and FAB mass data, the proposed structures for the complexes are shown in Figure 1.

**Redox properties**: Redox properties of the ruthenium complexes have been studied in DMF solution ($10^{-3}$M) in the potential range ±2V using $Ag/Ag^+$ as reference and graphite disc as working and $Ag/Ag^+$ as reference electrodes. Redox potential ($E°$) data are reported in Table III.

Table III: Electrochemical data of the Ru(III) complexes

| Complexes | Oxidations potential $E°$ (V) | Reductions potential $E°$ (V) |
|---|---|---|
| **1** $[Ru(L^1H)_3]3Cl^-$ | +1.15, +0.82 | -0.86, -1.37 |
| **2** $[Ru_2(L^2H_2)Cl_6(H_2O)_2]$ | +1.20 | -7.24, -1.40 |
| **3** $[Ru_2(L^3H_2)Cl_6(DMSO)_2]$ | +1.40, +1.20 | -0.86, -1.51 |
| **4** $[Ru_2(L^1H)(Hbbip)(DMSO)]\,(PF_6)_2$ | + 1.12 | -0.79, -1.44, -1.70 |
| **5** $[Ru_2(L^2H_2)(Hbbip)_2Cl_2](PF_6)_2$ | +1.10 | -0.72, -1.46, -1.80 |

- Obtained in DMF solution ($10^{-3}$M) containing 0.1 mol $dm^{-3}[Bu_4N]ClO_4$ as supporting electrolyte and potentials were determined with $Ag/Ag^+$ as reference and platinum wire as an auxiliary electrode at room temperature and 200 mV/s scan rate.

**Oxidation**

Cyclic-voltammogram of the mononuclear complex showed two irreversible oxidations at +0.82 and +1.15 V. Since free ligand also showed an oxidation peak at +1.00 V so distinction between ligand-based and metal-based oxidation was difficult. However in view of earlier report [24] the latter peak at 1.15 V could be considered to arise from Ru(III) → Ru(IV) oxidation further more dinuclear complex $[Ru_2L^3H_2Cl_6(DMSO)_2]$ showed two oxidation peaks at +1.40 and 1.20 V which could arise due to subsequent oxidation of two ruthenium centres.

Other complexes showed only one broad oxidation peak indicating that ligand based oxidation is overlapping with the metal-based oxidation.

**Reductions**

The complexes **1** - **3** showed two reduction peaks in the range -0.72 to -0.86V and -1.37 to 1.51 V whereas complexes **4** and **5** showed three reduction peaks in the range -0.72 to -0.79, -1.44 to -1.46V and -1.80 to -1.79V which were consistent with earlier report [25].

**Room temperature emission spectra**

Emission properties of the complexes have been studied in DMF solution ($10^{-5}$ M) at room temperature and the data are presented in Table II. The complexes **1** - **3** after excitation at 330 nm emitted at 460, 436 and 555 nm respectively indicating that the dinuclear complex **3** emitted at lower energy than the complexes **1** and **2**. The complexes **4** and **5** showed two emissions at 370, 719 and 375, 725 nm respectively. In view of an earlier report [26] two emissions in the range 370

- 375 and 719-720 nm were considered to arise probably from bridging ligand to metal and terminal ligand to metal charge-transfer. The better luminescence observed for the mononuclear complex **4** as compared to dinuclear complex **5** was also found to be consistent with the earlier report [27].

**Antibacterial studies**

The antibacterial activity of the ruthenium (III) complexes was evaluated against *Pseudomonas aeruginosa* and *E. coli* in DMSO solution ($10^{-5}$M) using the susceptibility testing method [28] as reported earlier [12]. Inhibitory effects by the free ligands ($L^1H$-$L^3H_2$) against *Pseudomonas aeruginosa* have been discussed earlier [12] by us. The data shown in Table IV indicate that the ruthenium complexes are more active against *E.coli* as compared to *Pseudomonas aeruginosa*.

Table IV: Antibacterial activity of Ru(III) complexes

| Compound | Zone of Inhibition (cm) | |
|---|---|---|
| | *Pseudomonas aeruginosa* | *E. coli* |
| $[Ru(L^1H)_3]3Cl^-$ | 0.25 | 0.55 |
| $[Ru_2(L^2H_2)Cl_6(H_2O)_2]$ | 0.19 | 0.50 |
| $[Ru_2(L^3H_2)\ Cl_6\ (DMSO)_2]$ | 0.30 | 0.75 |
| $[Ru(L^1H)(Hbbip)\ (DMSO)]\ (PF_6)_2$ | 0.22 | 0.60 |
| $[Ru(L^2H_2)(Hbbip)_2\ Cl_2]\ (PF_6)_2$ | 0.20 | 0.48 |

Concentration of samples was 10 μM in DMSO and inhibition zones were measured after subtracting the inhibition by the free solvent (DMSO) used as control.

**Antitumour and Anti-HIV studies**

The antitumour and anti-HIV activities were evaluated using standard procedures [29, 30]. The cytotoxicity data of free ligands ($L^1H$ - $L^3H_2$) alongwith their ruthenium complexes are shown in Table V. Among the free ligands ($L^1H$ - $L^3H_2$), the activity trend was observed to be $L^3H_2 > L^1H > L^2H_2$ indicating that the dinucleating ligand $L^3H_2$ was most active as compared to $L^2H_2$ against both tumour cells viz. A549 and U87-MG. However, the antitumour activity of ruthenium (III) complexes **1-5** was found to be significant as compared to their free ligands. The highest activity was shown by complex **3** which contains the most active ligand ($L^3H_2$) against both the tumour cells. The mononuclear complex **1** showed better activity as compared to dinuclear complex **2**. The better activity of complex **4** as compared to complex **1** against U87-MG was considered in terms of the presence of the bio-active benzimidazolyl group. The complex **5** did not show activity against any of both cells however it had a significant effect on the growth of only the glioblastoma cell line (clumping behaviour). A detailed mechanism of these activities is yet to be explored.

The anti-HIV activities (Table V) trend of the free ligands were again in the sequence $L^3H_2 > L^1H > L^2H_2$ indicating that the ligand $L^3H_2$ was the most active. However ruthenium (III)complexes showed a better activity as compared to the free ligands. The activity of these compounds was compared with the standard AZT (azido-thymidine) treated as control under the similar experimental conditions.

Table V: Antitumour and anti-HIV activities of free ligands and their Ru(III) complexes.

| Compound | Tumour cells | | Anti-HIV |
|---|---|---|---|
| | A549 | U87-MG | $IC_{50}$ (μg/mL) |
| $L^1H$ | >20 (16) | > 20 (9.1) | 22.00 |
| $L^1H_2$ | > 20 (10.2) | NA | 25.30 |
| $L^3H_2$ | 14.4 | 11.5 | 21.80 |
| **1** | > 20 (26.3) | NA | 20.7 |
| **2** | > 20 (17.4) | > 20 (10.3) | 21.4 |
| **3** | 13.5 | 13.00 | 19.7 |
| **4** | > 20 (11.6) | > 20 (21) | 23.3 |
| **5*** | NA | NA | 21.3 |
| AZT | - | - | 500 |

Values are $IC_{50}$ concentration in μg/mL. Percent inhibition observed is the value in brackets.
NA = Not active - inhibition < or = 5% at 20 μg/mL.
*The sample was not active but had a significant unique impact on growth behaviour of U87-MG cells at 20 and 10 μg/mL. AZT = Azido-thymidine

**Acknowledgement**
Authors thank to Prof. H. Itokawa, School of Pharmacy, University of North Carolina, USA for his help in evaluating the biological data. Financial assistance provided by Department of Atomic Energy (DAE, Mumbai) is also gratefully acknowledged.

**References**

1. P. Bernhard; A. Stebler; A. Ludi, *Inorg. Chem.*, 1984, **23**, 2151.
2. E.M. Kober and T.J. Meyer; *J. Am. Chem. Soc.*, 1983, **22**, 1614.
3. C. Daul, and A. Goursot,. *Inorg. Chem.*, 1985, **24**, 3554.
4. A.K. Mukherjee and A. Chakravorty; *Inorg. Chem.*, 1986, **25**, 1715.
5. G.K. Lahiri, S. Bhattacharya, B.K. Ghosh and A. Chakravorty, *Inorg. Chem.*, 1987, **26**, 4324.
6. E.C. Constable and A.M.W.C. Thompson, *J. Chem. Soc. Dalton Trans.*, 1992, 3467 and reference therein.
7. C. Piguet, J.C.G. Bunzli, G. Bernasdinelli, C.G. Bochet and P. Froidevaux, *J. Chem. Soc. Dalton Trans.*, 1995, 83.
8. S. Ptai, *The Chemistry of the hydrazo, azo and azoxy groups*, Part I, Wiley and Sons, New York (1975) Chapter-13.
9. V. Van, M. Paul, S.M.S. Toekimin, J.G. Haasnoot, J. Reedijk, O. Novakova, O. Vruna, V. Brabec, *Inorg. Chim. Acta*, 1995, **231**, 57.
10. M. Hartmann, T.J. Einkauser and B.K. Kepplar, *J. Chem. Soc.; Chem. Commun.*, 1996, 1741.
11. G. Sava, G. Salerno, A Bergamo, M. Cocchietto, R. Gagliardi, E. Alessio and G. Mestroni, *Metal Based Drugs*, 1996, **3**, 67.
12. L. Mishra, A.K. Yasaw, C.S. Choi and K. Araki, *Indian J. Chem.*, 1999, **38A**, 339.
13. L. Mishra, A.K. Yadaw, S. Srivastava and A.B. Patel, *New J. Chem.*, 2000, **24**, 505.
14. L. Mishra and A.K. Yadaw, *Indian J. Chem.*, 2000, **39A**, 660.
15. L. Mishra, A.K. Pandey and U.C. Agarwala, *Indian J. Chem.*, 1993, **37A**, 442.
16. D.T. Sawyer, A. Sobkowiak and J.J.L. Roberts, *Electrochemistry for Chemists*, 2nd edn., Wiley, New York (1995).
17. R.A. Krause, *Inorg. Chim. Acta*, 1977, **22**, 209.
18. A. Pacheco, B.R. James and S.J. Rettig, *Inorg. Chem.*, 1995, **34**, 3477.
19. E.C. Constable and A.M.W.C. Thompson, *J. Chem. Soc., Dalton Trans.*, 1992, 3467.
20. L. Mishra and R. Sinha. *Indian J. Chem*, **39A,** 2000 (In press).
21. G. Denti, S. Campagna, L. Sabatino, S. Serroni, M. Ciano and V. Balzani, *Photochemical conversion and storage of Energy*, (1991) p. 27.
22. M.M.T. Khan, N.H. Khan, R.T. Kureshy and A.B. Boricha, *Inorg. Chim. Acta*, 1990, 174, 175.
23. A.E.M. Ramadan and I.M.E. Mehasseb; *Transition, Met. Chem.*, 1997, **22**, 529.
24. G.K. Lahiri, S. Bhattacharya, M. Mukherjee, A.K. Mukherjee and A. Chakravorty, *Inorg. Chem.*, 1987, **26**, 3359.
25. B.K. Ghosh and A. Chakravorty, *Coord. Chem. Rev.*, 1989, **95**, 239.
26. T.E. Keyes, C.O. Connor and J.G. Vos, *J. Chem. Soc., Chem. Commun.*, 1998, 889.
27. S. Boyde, G.F. Strouse, W.E. Jones and T.J. Meyer, *J. Am. Chem. Soc.*, 1990, **112**, 7395.
28. C.V. Cervantes, J. Chavez, N.A. Cordova, P. Delamora and J.A. Velasco, *J. Microbios*, 1996, **48**, 159.
29. L.V. Rubinstein, R.H. Shoemaker, K.D. Paull, R.M. Simon, S. Tosini, P. Skehan, D.A. Schudiero, A. Monks and M.R. Boyd., *J. National Cancer Institute*, 1990, **82**, 1113.
30. P. Skehan, R. Storeng, D. Scudiero, A. Monks, J. McMohan, D. Vislica, T.J. Warren, H. Bokesh, S. Kenney and M.R. Boyd, *J. National Cancer Institute*, 1990, **82**, 1107.

**Received: January 11, 2001 – Accepted: February 7, 2001 – Accepted in publishable format: March 5, 2001**

